# Editorial: Biomarkers of mental well-being continuum across life span: from excelling to crisis

**DOI:** 10.3389/fphar.2023.1357630

**Published:** 2024-01-12

**Authors:** Kyoko Koshibu

**Affiliations:** Health, Nutrition and Care, DSM-Firmenich, Kaiseraugst, Switzerland

**Keywords:** mental well-being, mental health, happiness, depression, biomarker

We started this Frontiers Research Topic to better understand the existing biological evidence and biomarkers for happiness. This concept evolved into delineation of the mental wellbeing continuum. The absence of mental disorder is frequently used to define mental wellbeing. But it goes beyond that. It also refers to the ability to cope with daily stressors; to maintain happiness, health, and prosperity; and to make productive and fruitful contributions to one’s community (World Health Organization, 2022). As such, mental wellbeing exists in a complex continuum, with states of happiness and wellbeing at one end and debilitating mental health crises, such as burnout, anxiety, and depression, at the other. Maintaining mental wellbeing is no small task. The homeostasis is continuously challenged by stimulants (e.g., smoking and alcohol) and environmental factors (e.g., nutrition, exercise, and social support) (Dani and Harris, 2005; Tost et al., 2015; Smith and Merwin, 2021; Grajek et al., 2022). Individual differences in the ensuing psychological reactions make it even more difficult to determine the best course of action for each person in terms of both prevention and treatment. Consequently, a thorough comprehension of how these variables interact can aid in resolving the underlying heterogeneity of disease etiology in mental health disorders and can pave the way for novel approaches to individualized treatment or prevention.

The public realization of the importance of this individualized mental health continuum is reflected in the surge of personal health monitoring devices and biosensors in recent years (Kulkarni et al., 2022; Boiko et al., 2023; Seiferth et al., 2023). Managing mental wellness has been further ignited by the burdens of the COVID-19 pandemic, which increased the reported cases of anxiety and depression across different age groups and brought new concerns regarding its long-term consequences on mental health and performance (Leung et al., 2022; Zeng et al., 2023). Therefore, we decided to dedicate this Research Topic to an introduction to the biological evidence and biomarkers for mental wellbeing in order to increase awareness of the state of our knowledge about mental health and to gain a better understanding of how we could personalize mental health solutions.

The articles highlighted in the present Frontiers Research Topic can be divided into four subtopics: 1) biomarkers of mental health, 2) environmental factors affecting mental health, 3) nutritional or complimentary medicine for mental health and wellbeing, and lastly, the increasingly popular topic of 4) gut-brain functional connectivity for mental wellbeing. For biomarkers of mental health, Choi et al. offered another look at cholesterol as a biomarker of suicide as a part of the MAKE Biomarker discovery for Enhancing anTidepressant Treatment Effect and Response (MAKE BETTER) program conducted at Chonnam National University Hospital (Kang et al., 2018). In this *post hoc* analysis of a prospective study in South Korea, the authors found that the low cholesterol group was associated with increased suicidal severity and fatal/non-fatal suicide attempts in patients regardless of age. This finding is consistent with the low cholesterol hypothesis of mood disorders and suicide (Fiedorowicz and Haynes, 2010). The twist in this study was that when age was considered, the authors found that patients ≥60 years old in the high cholesterol group also showed increased suicidal severity, which resulted in a U-shape correlation between the cholesterol level and suicide attempts. Their findings therefore suggest that age-dependent analysis may be warranted. In a narrative mini review by Li et al., the authors explored the relationship between serotonin levels and depression in order to evaluate whether peripheral serotonin levels could be used as a biomarker for depression diagnosis and treatment. However, a clear correlation between peripheral serotonin levels and depression was not detected among the 33 studies evaluated. Thus, the authors concluded that the peripheral serotonin level may not be a good biomarker for depression diagnosis or antidepressant efficacy evaluation. Regarding the environmental effect on mental wellbeing, Ramos-Vera et al. provided a network analysis of the English Longitudinal Study of Aging (ELSA) COVID-19 sub-study, where the authors evaluated the mental health symptoms through online questionnaires and computer-assisted telephone calls in United Kingdom residents aged 50 years and above. Their findings suggest that depressive, anxious, and loneliness symptoms were dynamically affected by the pandemic context, such as the obligatory quarantine and vaccination efforts, in these older adults in the United Kingdom during the two waves of COVID in 2020. Regarding nutritional or complementary solutions to mental health, Zhang et al. summarized the latest evidence surrounding herbal medicine as an adjunctive therapy for adults with post-stroke depression in a meta-analysis of randomized controlled trials. In particular, the advantages of taking Shugan Jieyu capsules, Jie-Yu pills, or Wuling capsules with serotonin re-uptake inhibitors (SSRI) were highlighted. In yet another review by Cui et al., the complementary use of essential and volatile oils to maximize treatments for mood disorders as a validated method to treat depression, anxiety, and sleep in human and animal models was summarized. Similarly, the beneficial effects of vitamin B9 or folic acid on depressive- and anxiety-like behaviors were evaluated in a mouse model of postnatal immune activation (PIA) by Zhao et al., demonstrating the differential immune responses in astrocytes and microglia caused by folic acid. Last but not least, functional gut-brain connectivity was investigated by Hall et al. in patients with inflammatory bowel disease as a model of chronic inflammation. The authors demonstrated that Crohn’s disease and ulcerative colitis had differential effects on the resting spontaneous brain state dynamics in brain regions associated with the default-mode network and parietal and visual cortices, indicating that chronic gut inflammation is a key factor in the risk of developing abnormal brain network and behavior signatures, the effect of which is also related to the disease duration.

We are pleased to announce that at the time this Editorial was published, the top five countries that viewed the collection were the United States, China, Germany, Japan, and Ireland, indicating the global reach of the interest in mental wellbeing. We thank the authors who contributed to this Research Topic and encourage scientists around the world to continue the search for the biological basis of happiness and mental wellbeing.
